# Comprehensive analysis of culture-negative periprosthetic joint infection with metagenomic next-generation sequencing

**DOI:** 10.3389/fcimb.2025.1564488

**Published:** 2025-05-09

**Authors:** Lan Lin, Jiayu Li, Canhong Zhang, Juncheng Li, Baijian Wu, Zida Huang, Jianhua Lv, Mingzhong Liu, Wenbo Li, Wenming Zhang, Xinyu Fang

**Affiliations:** ^1^ Department of Orthopaedic Surgery, The First Affiliated Hospital of Fujian Medical University, Fuzhou, China; ^2^ Department of Orthopaedic Surgery, National Regional Medical Center, Binhai Campus of the First Affiliated Hospital, Fujian Medical University, Fuzhou, China; ^3^ Fujian Provincial Institute of Orthopedics, the First Affiliated Hospital, Fujian Medical University, Fuzhou, China; ^4^ Department of Orthopedic Surgery, Quanzhou First Affiliated Hospital of Fujian Medical University, Quanzhou, China; ^5^ Department of Orthopedic Surgery, Affiliated Hospital of Putian University, Putian, China

**Keywords:** periprosthetic joint infection, negative microbiological cultures, metagenomic next-generation sequencing, microbiology, risk factors

## Abstract

**Objective:**

This study aimed to identify the risk factors and microbial profiles of patients with culture-negative periprosthetic joint infection (PJI) using metagenomic next generation sequencing (mNGS) and to compare the clinical characteristics and treatment outcomes of culture-negative PJI (CN PJI) with culture-positive PJI (CP PJI).

**Methods:**

A retrospective analysis was conducted on 223 patients who met the International Consensus Meeting criteria for PJI and underwent surgical treatment at our hospital between February 2013 and January 2023. Clinical and follow-up data, including microbiological culture results and mNGS findings, were collected. Based on culture results, patients were divided into the CP PJI and CN PJI groups. Risk factors and microbial profiles of CN PJI patients were summarized with the aid of mNGS results. Differences in clinical characteristics and treatment outcomes between the two groups were also analyzed.

**Results:**

Among the 223 patients, 168 were in the CP PJI group, and 55 were in the CN PJI group. Risk factors for negative cultures included polymicrobial infections, infections caused by rare pathogens, and prolonged antibiotic use prior to sampling. In the CN PJI group, over a quarter of cases involved polymicrobial infections (25.5%) or rare pathogen infections (38.2%), with *Mycoplasma* sp. being the most frequently identified rare pathogen (7.2%). Compared to the CP PJI group, the CN PJI group exhibited distinctly longer hospital stays (*P*<0.001), extended antibiotic use (*P*=0.02), and a higher rate of antibiotic-related complications (*P*=0.026). However, no significant difference was noted in reinfection rates between the two groups (*P*=0.412).

**Conclusion:**

CN PJI presents a unique microbial spectrum and distinct clinical therapeutic characteristics. mNGS offers a more comprehensive understanding of infecting microorganisms, particularly those often missed by conventional culture techniques. With advancements in sample collection, optimized culture methods, molecular diagnostic tools, and early targeted therapies, CN PJI may achieve clinical outcomes comparable to CP PJI.

## 1 Introduction

With the aging population, the prevalence of severe joint degeneration has been steadily rising. Total joint replacement is widely recognized as an effective intervention to effectively improve the quality of life in patients with advanced joint diseases, alleviating pain and functional impairments associated with these conditions (Walker et al., 2002; Bumpass and Nunley, 2012). Despite these remarkable benefits, periprosthetic joint infection (PJI) remains a severe complication and the leading cause of failure in both primary and revision joint replacement surgeries (Bozic et al., 2010; Illingworth et al., 2013). The incidence of PJI is reported to range from 1-3% for primary joint replacements and 4-12% for revision surgeries (Della Valle et al., 2011; Kapadia et al., 2016), with a mortality rate as high as 20% within 5 years of diagnosis (Patel, 2023). Once it occurs, it not only leads to the involvement of bone and soft tissues (e.g., the patellar tendon), affecting joint function, but also imposes significant physical, psychological, and economic burdens on patients.

Accurate identification of the causative pathogen is essential for guiding effective, targeted antibiotic therapy for PJI. Currently, microbial culture of infected tissue and synovial fluid is deemed as the gold standard for pathogen diagnosis. However, studies have documented that microbial cultures yield negative results in up to 42% of PJI cases (Tan et al., 2018; Goswami et al., 2022). In such culture-negative PJI (CN PJI) cases, the inability to identify the pathogen poses a significant challenge for clinicians, making it difficult to provide targeted antimicrobial therapy. This lack of targeted treatment is a major concern, as CN PJI patients are reported to have recurrence and reoperation rates more than 4 times higher than those of patients with CP PJI (Mortazavi et al., 2011; Goswami et al., 2022). The recurrence of CN PJI involves the following primary mechanisms: (1) Pathogen persistence via biofilm formation on implant surfaces, which shields bacteria from host immune defenses and antibiotic clearance, thereby promoting chronic/recurrent infections (Van Roy et al., 2024); (2) Prolonged or inappropriate broad-spectrum antibiotic use disrupts commensal microbiota homeostasis (dysbiosis), thus enhancing host susceptibility to colonization by opportunistic pathogens (Jandl et al., 2024). PJI is a complex infectious disease, and the occurrence of CN PJI is influenced by multiple factors. One critical factor is the prolonged use of antibiotics before sampling, which has been shown to distinctly increase the likelihood of culture-negative results (Berbari et al., 2007). Other contributing factors include infections caused by low-virulence microorganisms, biofilm formation, insufficient sample volume, and improper or delayed transportation of specimens (Goswami and Parvizi, 2020).

The rapid advancement of molecular diagnostics has significantly improved the detection of pathogenic microorganisms in complex infectious diseases. Quantitative polymerase chain reaction (qPCR) facilitates precise microbial identification utilizing primers that are complementary to specific DNA sequences of target species. Furthermore, early studies have demonstrated the high sensitivity of qPCR in detecting microorganisms within biofilms (Stoodley et al., 2011; Jacovides et al., 2012). However, the primary limitation of qPCR lies in its inability to detect pathogens beyond a predetermined range, which greatly restricts its utility in diagnosing CN PJI (Chiu and Miller, 2019; Diao et al., 2022). Metagenomic next-generation sequencing (mNGS), on the other hand, is a high-throughput, unbiased screening method that combines advanced sequencing technology with bioinformatics analysis to comprehensively identify all known microorganisms (including bacteria, fungi, viruses, and parasites) in clinical samples (Fang et al., 2020). This revolutionary approach broadens the scope of molecular diagnostic techniques, offering unprecedented value in diagnosing infectious agents, particularly in challenging cases of CN PJI (Tarabichi et al., 2018a; Tarabichi et al., 2018b; Goswami et al., 2022).

To date, reports on a comprehensive analysis of CN PJI based on larger sample sizes are scarce. This study focused on this gap by analyzing the clinical data of a substantial cohort of CN PJI patients treated at our center. Utilizing molecular diagnostic techniques such as mNGS, we conducted an integrated analysis of the potential risk factors, microbial profiles, and clinical treatment characteristics of CN PJI. Our goal is to provide surgeons with deeper insights into the diagnostic and therapeutic nuances of CN PJI.

## 2 Materials and methods

### 2.1 Patient selection

With the approval of the Ethics Committee of the First Affiliated Hospital of Fujian Medical University (MRCTA, FMU ECFAH 2018 [026]), we retrospectively collected clinical data from 223 PJI patients who underwent revision surgery at our center between February 2013 to January 2023. All patients underwent joint replacement surgery prior to developing PJI, including 94 total knee arthroplasty (TKA), 14 unicompartmental knee arthroplasty (UKA), 84 total hip arthroplasty (THA), and 31 hip hemiarthroplasties. Upon admission, all patients underwent a comprehensive evaluation, which included serum inflammatory markers [white blood cell (WBC) count, erythrocyte sedimentation rate (ESR), C-reactive protein (CRP), synovial fluid polymorphonuclear neutrophil and synovial fluid WBC (SF-WBC)]. The diagnosis of PJI was validated through at least two senior orthopedic surgeons, two senior infectious disease specialists, and one senior microbiologist in accordance with the criteria outlined by the Musculoskeletal System Infection Association (Parvizi et al., 2011). Inclusion criteria were: 1. patients diagnosed with PJI; 2. patients who provided informed consent; 3. patients with complete clinical data. The exclusion criteria were: 1. patients with comorbid malignant tumors were excluded to avoid immune dysfunction or treatment-related biases; 2. those with other active infections were excluded to isolate inflammatory and microbiological profiles specific to PJI; 3. culture-negative and mNGS-negative or without mNGS testing were excluded to ensure all included PJIs had definitive pathogen identification; 4. follow-up period of less than 2 years.

### 2.2 Specimen collection and microbial culture

Periprosthetic tissue samples were collected from five different sites and minced. Tissue homogenates were prepared by an automatic high-speed tissue homogenizer (JXFSTPRP24, Jingxin Industrial, Shanghai, China) and inoculated onto Columbia blood agar plates (HBPM0124-15, Haibo Biotechnology, Qingdao, China) under both anaerobic and aerobic conditions. For liquid specimens (synovial fluid, wound exudate, etc.), the specimens were directly introduced into BACTEC Plus aerobic (442192, Becton-Dickinson, Franklin Lakes, New Jersey, USA) and anaerobic vials (442193, Becton-Dickinson, Franklin Lakes, New Jersey, USA) and incubated in an automated thermostat (Bactec 9050, Becton-Dickinson, Franklin Lakes, New Jersey, USA) at 37°C with 6% CO_2_ for 14 days. Prosthetic components removed during surgery (such as knee pads, hip liners, etc.) were placed in 400 ml of sterile saline and treated with ultrasonic vibration (40 Hz, 5 min) to disrupt the biofilm and generate ultrasonic lysate. The lysate was centrifuged, and the supernatant was discarded. The resulting sediment was pelleted by centrifugation at 10,000 x g for 15 minutes at 4°C. The resulting sediment was inoculated into aerobic culture bottles (442192, Becton-Dickinson, Franklin Lakes, New Jersey, USA) and anaerobic culture bottles (442193, Becton-Dickinson, Franklin Lakes, New Jersey, USA) for incubation under the same conditions (37°C, 6% CO_2_, 14 days).

### 2.3 mNGS with results interpretation

Specimen pre-treatment was consistent with that described in microbiological culture and mNGS was performed following the protocol described previously (Huang et al., 2020). Briefly, DNA was extracted from the samples utilizing the TIANamp Micro DNA kit (DP316, Tiangen, China), which was completely fragmented into small fragments of about 200–300 bp in size. Rolling circle amplification was then used to replicate DNA, forming DNA nanospheres. These nanospheres were loaded onto the sequencing chip and sequenced utilizing the BGISEQ-500 platform (UWIC, China). The human genome sequence (Hg19) was filtered out utilizing Burrows-Wheeler alignment, and the remaining sequencing data were compared with microbial databases for taxonomic classification of microorganisms.

The definition of rare pathogens was based on previous reports (Anagnostakos et al., 2021; Lyu et al., 2024). For cases where microbial cultures were negative but mNGS results were positive, the following criteria were adopted to test whether the mNGS result represented a “true positive” (Ding et al., 2024): 1. The pathogen identified by mNGS has been previously reported to cause bone and joint infections, and the clinical characteristics are consistent with such infections; 2. A third diagnostic method, such as 16S rRNA sequencing, confirms the same pathogen identified by mNGS. For the 16S rRNA sequencing technique, briefly, we extracted total microbial DNA from synovial fluid, sonicate prosthetic fluid, and tissue homogenate specimens using the TIANamp Micro DNA Kit (CDP316, Tiangen Biotech, China). PCR amplification targeting the V4 region of the bacterial 16S rRNA gene was performed in a 25 μl reaction volume with the TopTaq DNA Polymerase kit (AP151-13, TransGen Biotech, China), using primers 515f (5′-GTGCCAGCMGCCGCGGTAA-3′) and 806r (5′-GGACTACHVGGGTWTCTAAT-3′); 3. Targeted antibiotic therapy is determined to be effective by at least 3 senior clinicians.

### 2.4 Surgical method selection

All patients were treated according to the Tsukayama classification system (Hulleman et al., 2023). Briefly, for patients with early postoperative PJI or acute hematogenous dissemination of PJI (Tsukayama Type II/III), debridement with implant retention (DAIR) was typically adopted. For patients with Tsukayama Type I/IV PJI, without sinus tract formation and infections caused by non-multi-resistant organisms, a one-stage revision was chosen. In chronic PJI patients (chronic Tsukayama Type IV) with sinus tract formation, multidrug-resistant pathogens, and poor soft tissue coverage, a two-stage revision was generally preferred. Patient preferences also played a role in determining the final surgical strategy. The surgical procedures were implemented by a consistent treatment team at our center.

### 2.5 Follow up

Patients were followed up every 3 months through outpatient visits and telephone calls for at least 2 years, with reinfection and mortality as the primary endpoints. Regular reviews were conducted for infection-related serum inflammatory markers, such as ESR and CRP. Infection control was assessed based on the Delphi consensus (Diaz-Ledezma et al., 2013). Infection control was defined as the successful completion of treatment without infection-related complications or the need for reoperation. This definition ensures long-term eradication of the infection, rather than merely transient suppression.

Antibiotic-related complications were defined as follows (Xu et al., 2022): 1. Myelosuppression: a WBC count above 4×10^9^/L prior to antibiotics, but below 3×10^9^/L during treatment; 2. Hepatic impairment: Elevation of hepatic function indexes [alanine aminotransferase (ALT) and aspartate aminotransferase (AST)] by more than 1.5 times the baseline levels prior to antibiotic administration; 3. renal impairment: An increase in serum creatinine by more than 1.5 times the normal baseline level before antibiotic use; 4. Gastrointestinal symptoms: Severe nausea, vomiting, and dyspepsia indicative of antibiotic-induced gastrointestinal disturbances. These laboratory results, along with clinical symptoms, were considered indicative of adverse effects on organ function due to antibiotic use.

### 2.6 Statistical analysis

Continuous variables were first studied for normality utilizing the Kolmogorov-Smirnov test. Normally distributed variables are summarized as mean ± standard deviation, and comparisons were made utilizing the independent-samples t-test. Non-normally distributed variables are reported as median (interquartile range), with the Mann-Whitney U test adopted for comparisons. Categorical variables were compared between groups employing the Chi-squared test or Fisher’s exact test, as appropriate. Binary logistic regression analysis was performed to identify potential risk factors based on demographic data. Infection control during the follow-up period was assessed in both groups employing the Kaplan-Meier survival analysis. All statistical analyses were made utilizing SPSS 26.0 (IBM, USA), and *P* < 0.05 signified statistically significant.

## 3 Results

### 3.1 Demographic characteristics

**Figure 1:**
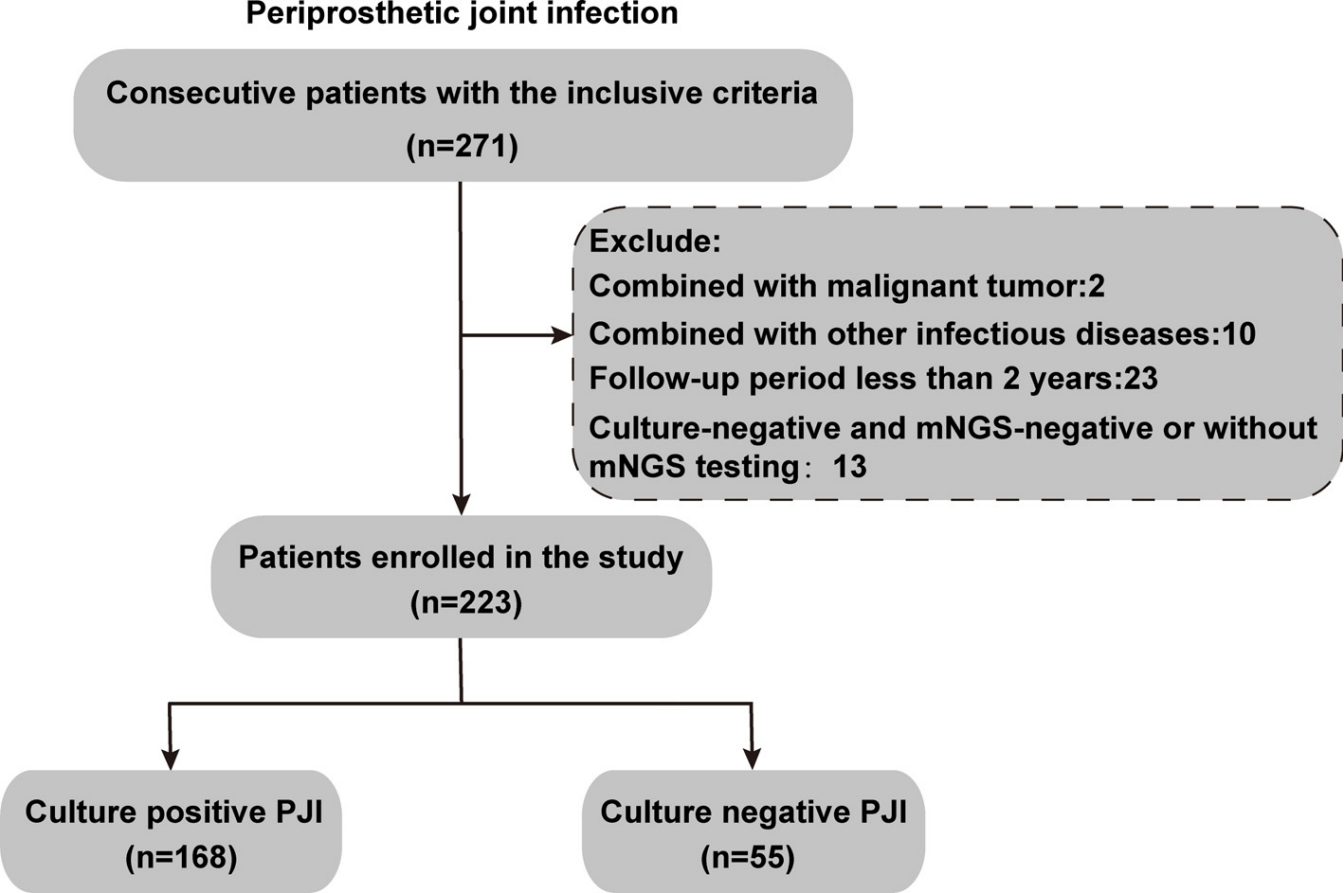
Flow chart of the inclusion, exclusion, and grouping of PJI cases in this study.

Our cohort consisted of 271 patients who met the 2018 International Consensus Meeting criteria (Parvizi et al., 2018) for PJI and were hospitalized at the First Hospital of Fujian Medical University between February 2013 and January 2023. After applying the exclusion criteria, 223 patients were finally included (**Figure 1**). Based on the results of microbiological cultures and mNGS of synovial fluid, prosthetic ultrasound fluid, and intraoperative tissue samples, 168 culture-positive patients were assigned to the CP PJI group, and 55 patients with negative routine cultures but positive mNGS results were included in the CN PJI group. The baseline characteristics of the two groups were similar, with no significant differences in age, gender, BMI, surgical site, Charlson comorbidity index (CCI), the presence or absence of preoperative invasive procedures, sinus tract formation, or preoperative serological tests (**Table 1**). However, relative to the CP PJI group, the CN PJI group had a higher proportion of multiple infections (*P*=0.042), infections caused by rare pathogens (*P*<0.001), and a longer duration of antibiotic use prior to sampling (*P*<0.001) (**Table 1**). These findings suggest that multiple infections, infection with rare pathogens, and prolonged antibiotic use prior to sampling may notably influence the results of microbial cultures.

**Table 1:** Comparison of characteristics between culture positive and culture negative groups.

Characteristics	CP PJI (n=168)	CN PJI (n=55)	P-value
Age (years)	65.46 ± 11.2	68.00 ± 11.8	P=0.153
Gender (male/female)	72/96	26/29	P=0.567
BMI (kg/m^2^)	24.56 ± 3.19	24.06 ± 3.09	P=0.310
Joint (hip/knee)	88/80	27/28	P=0.672
CCI	2.48 ± 1.35	2.82 ± 1.38	P=0.108
PIP (y/n)	112/56	41/14	P=0.271
Sinus tract (y/n)	42/126	17/38	P=0.389
Multiple infection (y/n)	22/144	15/40	P=0.016*
Rare pathogen infection (y/n)	18/150	21/34	P<0.001*
Prior use of antibiotics (days)	3.43 ± 4.17	8.16 ± 5.80	P<0.001*
Laboratory data			
WBC (×10^9^/l) (IQR)	6.63 (5.39,8.65)	6.44 (4.65,8.86)	P=0.468
ESR (mm/h) (IQR)	63.50 (40.00,89.75)	41.00 (30.00,82.00)	P=0.062
CRP (mg/l) (IQR)	34.85 (12.40,67.35)	19.83 (11.60,45.47)	P=0.059
SF WBC (/ml) (IQR)	10914.00 (4552.00,35429.50)	12892.00 (3781.00,27391.00)	P=0.790
SF PMN (%) (IQR)	77.80 (67.00,89.48)	74.80 (51.10,88.20)	P=0.051

CP, culture positive; CN, culture negative; BMI, body mass index; CCI, Charlson comorbidity index; PIP, preoperative invasive procedures; WBC, white blood cell count; CRP, C-reaction protein; ESR, erythrocyte sedimentation rate; SF, synovial fluid; PMN, polymorphonuclear neutrophils; IQR, interquartile range.

### 3.2 Risk factors for CN PJI

**Figure 2:**
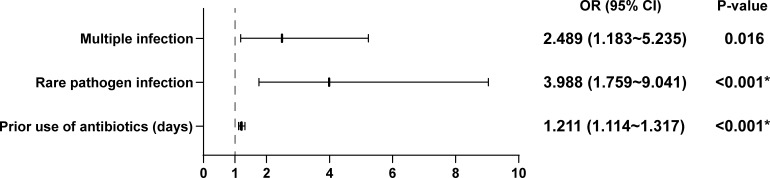
Binary logistic regression analysis of risk factors associated with negative microbiological culture results in PJI.

Based on the findings illustrated in **Table 1**, we performed multivariate logistic regression to examine the impact of multiple infections, rare pathogen infections, and the duration of antibiotic use prior to sampling on the likelihood of negative microbiological cultures. The results demonstrated that multiple infections were linked to a 1.489-fold increase in the probability of negative microbiological cultures in PJI patients (OR=2.489, 95%CI [1.183-5.235], *P*=0.016). Patients infected with rare pathogens had a nearly fourfold increased risk of negative cultures compared to those infected with non-rare pathogens (OR=3.988, 95%CI [1.759-9.041], *P*<0.001). Additionally, each additional day of antibiotic use prior to sampling was linked to a 21.7% increased likelihood of a negative culture result (OR=1.211, 95%CI [1.114-1.317], *P*<0.001) (**Figure 2**).

### 3.3 Microbial profiles of CP PJI

In patients with culture-positive prosthetic joint infections (CP PJI), microbial cultures identified a total of 47 pathogens. The most common bacteria were *Staphylococcus aureus* (19.4%) and *Staphylococcus epidermidis* (20.3%) (**Supplementary Table 1**), with Gram-negative bacteria detected in 14.8% of cases. Among the culture-positive patients, *Staphylococcus aureus* was categorized into methicillin-sensitive *Staphylococcus aureus* (MSSA) and methicillin-resistant *Staphylococcus aureus* (MRSA), accounting for 12.9% and 6.5% of the total, respectively. *Staphylococcus epidermidis*, another prevalent pathogen, was detected in 20.3% of cases. Among the Rare pathogens, *Enterococcus faecalis* had a relatively higher detection rate, found in 1.8% of patients. Other frequently detected Gram-positive bacteria included *Staphylococcus capitis* (3.2%), *Staphylococcus haemolyticus* (3.7%), and *Staphylococcus hominis* (0.9%). In terms of Gram-negative bacteria, *Klebsiella pneumoniae* and *Escherichia coli* were present in 4.1% and 3.7% of cases, respectively. Additionally, *Pseud*omonas aeruginosa and Enterobacter cloacae were detected in some patients. In addition, 13.1% (22/168) of CP PJI were infected with two or more pathogens. A median of 3 and a mean (and standard deviation) of 3.2 ± 0.8 different pathogens were identified per case. Moreover, in 77.3% (17) of the 22 polymicrobial cases, multiple species defined as common (on the basis of a 5% incidence threshold) were present. *Staphylococcus aureus* and *Staphylococcus epidermidis* continued to be the pathogenic microorganisms with the highest frequency of occurrence in these patients with multiple infections.

### 3.4 Microbial profiles of CN PJI

**Figure 3:**
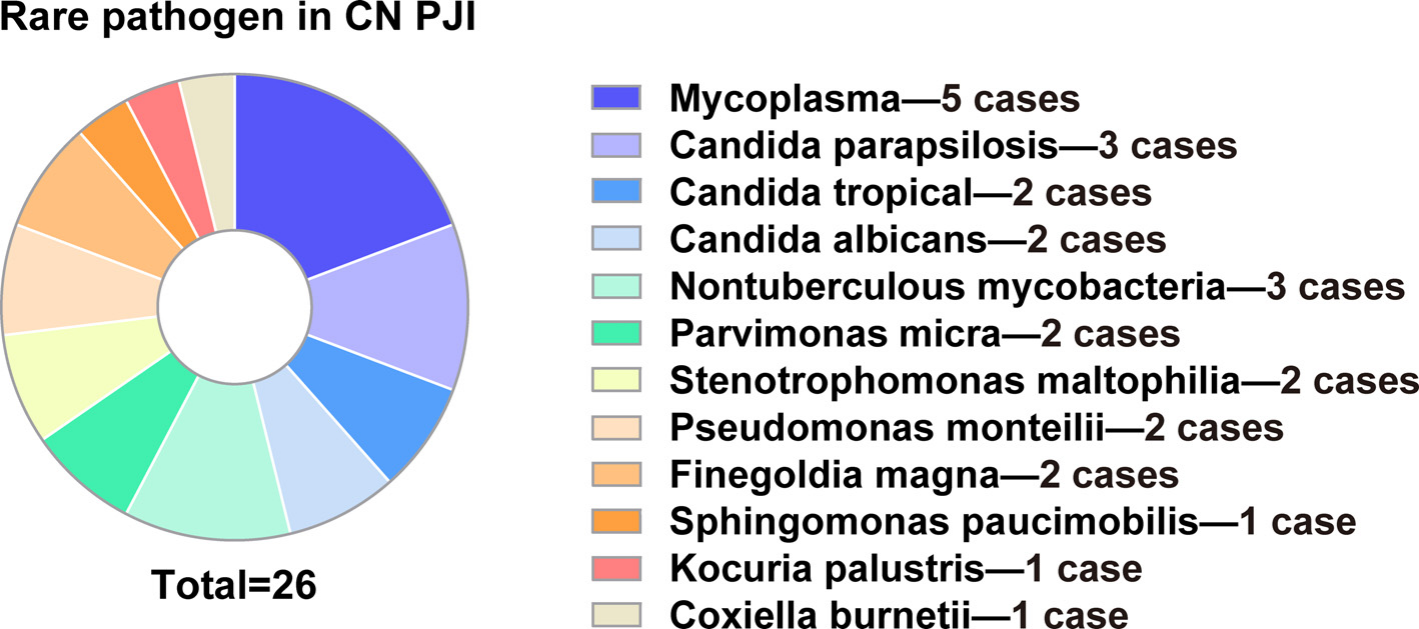
Distribution of various rare pathogens in culture-negative PJI cases.

mNGS testing was performed on samples collected from infected sites in PJI patients with negative microbiological cultures. Among these samples, prosthetic ultrasound fluid accounted for the largest proportion (37.18%), followed by joint fluid (33.21%) and fresh tissue (29.61%) samples. In the entire CN PJI cohort, 69 pathogens were detected by mNGS, with the most common organisms being *Staphylococcus epidermidis* (13.0%) and *Staphylococcus aureus* (11.6%) (**Supplementary Table 1**). Rare pathogen infections were identified in 38.2% of CN PJI patients, with 26 rare pathogens identified. The most common rare pathogens were *Mycoplasma* sp. (5, 7.2%), *Candida parapsilosis* (3, 4.3%) and *Nontuberculous mycobacteria* (3, 4.3%) (**Supplementary Table 1**) (**Figure 3**). The NTM species identified in this study comprised Mycobacterium colombiense (n=1), Mycobacterium abscessus (n=1), and Mycobacterium *arupense* (n=1), highlighting the diversity of atypical pathogens in culture-negative infections. In addition, 27.3% (15/55) of CN PJI were infected with two or more pathogens. A median of 2 and a mean (and standard deviation) of 2.1 ± 0.3 different pathogens were identified per case. In 66.7% (10) of the 15 polymicrobial cases, multiple species defined as common (on the basis of a 5% incidence threshold) were present. The mNGS results showed that *Staphylococcus aureus* and *Staphylococcus epidermidis* were not only the pathogenic microorganisms with the highest frequency in CN PJI with multiple infection, but also with the highest mean relative abundance.

### 3.5 Comparison of clinical variables

**Figure 4:**
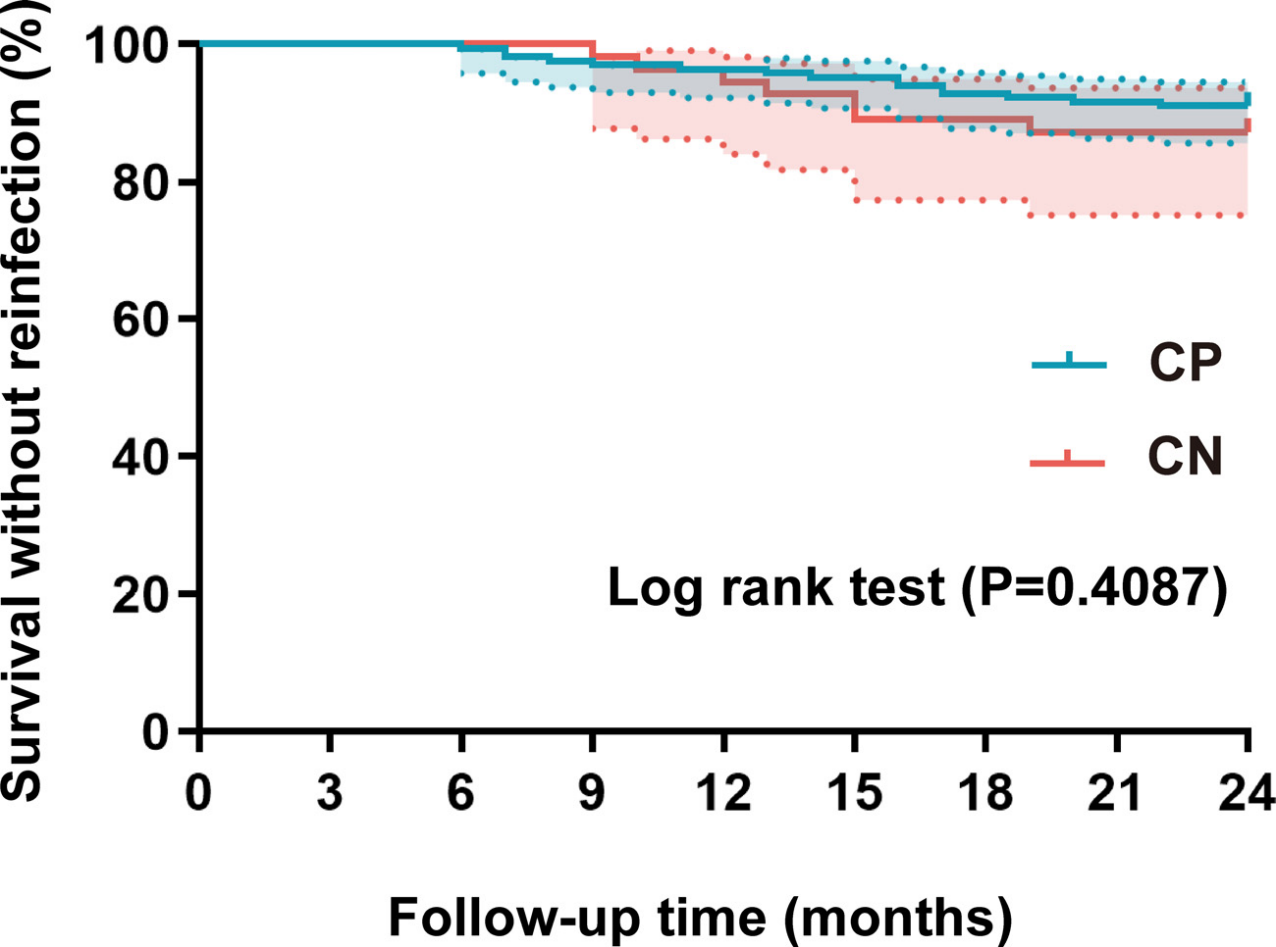
Kaplan-Meier survival curve analysis comparing reinfection rates between the two groups. The shaded areas denote 95% CIs. CP, culture positive; CN, culture negative.

No significant difference was noted concerning follow-up duration (*P*=0.251) or surgical approach (*P*=0.781) between the two groups. However, the median length of hospital stay was longer in the CN PJI group relative to the CP PJI group (*P <*0.001). The duration of antibiotic treatment was also longer in the CN PJI group (*P*=0.02), and the incidence of antibiotic complications was higher in this group as well (*P*=0.026). The one-year reinfection rate was 3.6% in the CP PJI group and 5.5% in the CN PJI group. These rates increased to 8.9% and 12.7%, respectively, over the two-year follow-up period. Most importantly, no notable difference was witnessed in the infection recurrence rate between the two cohorts during the 2-year follow-up, as assessed by Kaplan-Meier analysis (*P*=0.412) (**Table 2**) (**Figure 4**).

**Table 2:** Comparison of outcomes between culture positive and culture negative groups.

Variables	CP PJI (n=168)	CN PJI (n=55)	P-value
Follow-up period (months)	40.96 ± 15.67	43.76 ± 15.56	P=0.251
Treatment, n (%)			P=0.781
DAIR	27 (16.1%)	7 (12.7%) 16 (29.1%) 32 (58.2%)	
One-state revision	43 (25.6%)		
Two-state revision	98 (58.3%)		
Length of hospital stay, days (IQR)	18 (15,22)	22 (18,30)	P<0.001*
Duration of antibiotic use, days (IQR)	96 (61,112)	101 (83,134)	P=0.02*
Antibiotic complications (y/n)	19/147	13/42	P=0.026*
Reinfection (y/n)	15/153	7/48	P=0.412

CP, culture positive; CN, culture negative; DAIR, debridement with implant retention; IQR, interquartile range.

## 4 Discussion

Targeted antibiotic therapy is crucial in the management of PJI, especially in cases of CN PJI. Inadequate or ineffective antibiotic selection can lead to delayed treatment response or even treatment failure, highlighting the critical significance of early and accurate pathogen diagnosis. Recent studies have underscored the significant value of molecular diagnostic techniques, particularly mNGS, in guiding effective antimicrobial therapy (Han et al., 2019; Wang et al., 2020). mNGS provides a comprehensive microbiological profile that may not be detected by conventional culture methods (Goswami and Parvizi, 2020). Additionally, although mNGS is more expensive than conventional culture methods, its ability to provide rapid and comprehensive pathogen detection may reduce overall healthcare costs by enabling timely and targeted antimicrobial therapy (Pan et al., 2024). This, in turn, could potentially decrease the duration of hospitalization and the need for broad-spectrum antibiotics, contributing to better patient outcomes and lower treatment costs. Another key advantage of mNGS is its ability to detect microbial nucleic acids even when organisms are no longer viable due to prior antibiotic exposure (Langelier et al., 2018). This is particularly relevant in clinical settings where prolonged antibiotic use before sampling can lead to false-negative cultures. While culture relies on bacterial viability, mNGS can identify pathogens from residual DNA/RNA fragments, improving diagnostic yield in pretreated patients. In our study, we analyzed microbiological culture and mNGS results from 223 PJI cases (including 55 CN PJIs) at our center. A comprehensive analysis of the risk factors, microbiological profiles, and clinical treatment characteristics of CN PJI was conducted. Our findings revealed that multiple infections, infections with rare pathogens, and the duration of antibiotic use prior to sampling were key risk factors associated with microbiological culture-negativity. Recent studies have similarly demonstrated that mNGS is more tolerant than microbial culture to pre-sampling antibiotic use. When antibiotics have been administered for >3 days, the detection rate of mNGS is significantly higher than that of conventional culture (Ding et al., 2024). For atypical pathogens such as mycobacteria and fungi, culture often requires specialized growth media and stringent conditions, otherwise yielding negative results. Moreover, infections caused by these rare pathogens often present with distinctive clinical features (e.g., prior invasive procedures, sinus tracts, and high Charlson Comorbidity Index scores) (Lyu et al., 2024). Therefore, in patients with a history of prolonged antibiotic therapy, clinical features suggestive of rare pathogen infection, suspected polymicrobial infections, or chronic PJI with suspected biofilm formation, we should maintain a high index of suspicion for culture-negative results. In such cases, while performing conventional cultures, mNGS should also be conducted to identify pathogens that may be missed by culture. For patients with CN PJI and positive mNGS results, systematic treatment should be initiated. First, the risk of false-positive mNGS results must be carefully evaluated. Subsequently, targeted antibiotic therapy should be selected based on the mNGS findings. Despite the relatively prolonged antibiotic treatment and higher incidence of complications in CN PJI, targeted therapy based on mNGS results achieved success rates comparable to those observed in CP PJI cases.

The diagnosis of pathogens can be particularly challenging in cases involving multiple infections (Hoffman et al., 2006; Ding et al., 2024). In the present study, multiple infections were detected in more than a quarter (25.5%) of CN PJI based on mNGS results. Previous research has shown that in infections associated with orthopedic surgery, the relatively limited nutrient and spatial resources of microbial ecosystems often result in the dominance of two or more microorganisms, which can interfere with the normal colonization and growth of other coexisting flora (Goswami et al., 2022; Xie et al., 2023). This competitive interaction, especially in complex infections like PJI, may result in a reduced microbial load, complicating the isolation of all pathogens utilizing conventional culture methods. When microbial abundance falls below the sensitivity threshold for culture, the likelihood of negative culture results increases. Recent studies have also shown that multiple infection PJI may promote a viable but nonculturable (VBNC) state in microorganisms, further contributing to negative culture outcomes (Goswami et al., 2022).

Our comparative analysis revealed distinct microbial profiles between CP PJI and CN PJI groups, with important clinical implications. While *Staphylococcus aureus* (19.4% vs. 11.6%) and *Staphylococcus epidermidis* (20.3% vs. 13.0%) remained predominant in both groups, the CN PJI cohort demonstrated a 2.8-fold higher incidence of rare pathogens compared to CP PJI (38.2% vs. 10.7%). Notably, *Mycoplasma* sp*ecies* (7.2%) and *non-tuberculous mycobacteria* (4.3%) were exclusively detected in CN PJI cases through mNGS, organisms notoriously resistant to conventional culture due to their fastidious growth requirements. This disparity may explain the observed differences in initial treatment strategies—the CN PJI group’s prolonged antibiotic duration (*P*=0.02) likely reflects attempts at empirical coverage for these culture-elusive pathogens. Interestingly, gram-negative pathogens showed distinct distribution patterns. Although *Escherichia coli* was exclusively cultured in CP PJI (3.7%), CN PJI cases revealed unexpected gram-negative organisms like *Stenotrophomonas maltophilia* (2.9%) and *Pseudomonas monteilii* (2.9%), which are frequently associated with biofilm formation and antibiotic resistance. This microbial diversity in CN PJI aligns with the higher complication rates observed (*P*=0.026), as these organisms often require specific antimicrobial regimens not typically included in empirical protocols. Another key factor leading to negative microbiological cultures in PJI is infection with rare pathogens (Anagnostakos et al., 2021; Huang et al., 2023; Lyu et al., 2024). Consistent with previous studies, the most common microorganisms identified in our CN PJI cohort were *Staphylococcus epidermidis* (13.0%) and *Staphylococcus aureus* (11.6%) (Goswami et al., 2022). In addition, more than one-third (38.2%) of CN PJI cases in this cohort were linked to rare pathogens, a notably higher proportion than in the CP PJI group. Routine cultures have been shown to identify common microorganisms in up to 73.8% of cases, but culture positivity for rare pathogens is much lower, at just 53.1% (Lyu et al., 2024). Among the rare pathogens, *Mycoplasma* sp. was the most frequently detected in our cohort. *Mycoplasma* sp. is a common colonizer of the respiratory and genitourinary tracts and can cause PJI following invasive procedures, such as catheterization. The challenging culture conditions for *Mycoplasma* sp., including the need for specialized media and a prolonged incubation period of up to 3 weeks (Rieber et al., 2019), combined with the relatively low incidence of *Mycoplasma* sp. PJI, often result in missed diagnoses. Similarly, while *non-tuberculous mycobacteria* (NTM) can grow under routine culture conditions, they are slow-growing and require prolonged incubation times to be detected (Forbes et al., 2018). As a result, culture-positive results for NTM are difficult to obtain unless NTM infection is specifically suspected. As a high-throughput and highly sensitive sequencing technology, mNGS is invaluable in identifying pathogens in rare PJI cases. For atypical pathogens (*Mycoplasma* sp., NTM or fungi, etc.), in previous studies, our center proposed a standardized and optimized diagnostic protocol for mNGS. This protocol was refined through the following measures: (1) Optimization of culture conditions (e.g., extending incubation periods to 3 weeks and employing SP4 media for Mycoplasma spp. based on preliminary mNGS findings); (2) Integration of pathogen abundance thresholds with clinical symptom assessment (localized erythema, systemic inflammatory responses) to exclude low-abundance contaminants; (3) Implementation of iterative validation through repeated 16S rRNA sequencing or culture for cases with discordant mNGS results and clinical manifestations (Zhang et al., 2022; Cai et al., 2023).

In cases of CN PJI, the lack of reliable pathogen information at the early stage often leads clinicians to resort to broad-spectrum antibiotics or combination therapy, such as non-targeted treatment with antibiotics like vancomycin and meropenem. However, as shown in this study, over one-third of CN PJI patients had infections caused by rare pathogens, which are often resistant to coverage by broad-spectrum antibiotics. This resistance increases the risk of adverse events (Xiang and Lu, 2019). For example, *Mycoplasma* sp., which lacks a cell wall, is not susceptible to vancomycin, but is effectively treated with tetracyclines or quinolones (Wang et al., 2021). Similarly, NTM requires targeted anti-mycobacterial therapy, and their strong biofilm-forming ability makes it more resistant to antibiotics once a biofilm is established (Park et al., 2014). Although the delay in obtaining sufficient pathogen information early on initially hampers disease management, a high clinical success rate was finally achieved after using mNGS for pathogen identification and administering targeted antibiotics. Therefore, we recommend performing mNGS as early as possible when confronted with high-risk PJI cases that yield negative culture results. For example, preoperative synovial fluid can be tested utilizing mNGS to obtain comprehensive pathogen data early on (Fang et al., 2021), which can then be verified through optimized culture methods or 16S rRNA sequencing. Although this approach may increase treatment costs, it provides significant value by reducing adverse events and improving treatment success rates.

Despite the valuable findings of this study, there are several limitations that should be acknowledged. First, as a single-center retrospective study, it is subject to biases, despite enabling comparative analysis. Specifically, in cases of CN PJI, the decision to choose subsequent treatments based on culture or mNGS results could introduce selection bias, which may affect the final comparison of clinical outcomes. Second, due to sample selection bias and the varying sensitivity of mNGS across different sample types, the results of mNGS could differ depending on the sample analyzed. We also classified microorganisms with the highest abundance as the dominant flora, but when multiple samples from the same patient yield different results, the interpretation of these findings requires guidance from experienced microbiologists, which may introduce further bias. Third, this study excluded cases of PJI with negative results from both molecular diagnostic methods, such as mNGS, and conventional culture. However, clinicians frequently encounter cases where no effective pathogen information is obtained, which can lead to treatment failure. Therefore, further research into the risk factors, clinical characteristics, and treatment outcomes of such patients is of significant clinical importance. Finally, this study mainly addressed the mid-term treatment outcomes of CN PJI and CP PJI patients who received mNGS-based targeted therapy. Future studies should extend the follow-up period to determine potential disparities in long-term clinical outcomes between the two cohorts. These issues warrant further investigation through multi-center, completely randomized trials and extended clinical follow-up to address them comprehensively.

## 5 Conclusions

Overall, CN PJI represents a distinct subset of infections with unique risk factors, microbial profiles, and clinical management considerations. Specifically, our study identified multiple infections, the presence of rare pathogens, and the duration of antibiotic use prior to sampling as key risk factors for CN PJI based on mNGS results. Although negative culture results often lead clinicians to initially treat with broad-spectrum antibiotics, resulting in prolonged antibiotic use, subsequent targeted therapy based on mNGS results does not show a significant difference in infection control rates when compared to CP PJI, which is diagnosed and treated according to the “gold standard” culture method. Given these findings, clinicians should tailor microbiological treatment strategies to the individual patient’s circumstances. In cases of CN PJI, we recommend the integration of molecular diagnostics, such as mNGS, to optimize culture methods, facilitate early identification of causative organisms, and enable the timely development of targeted therapeutic strategies. This approach can help avoid the pitfalls of empirical treatment when the causative organism is unidentified. Nonetheless, further research is needed to determine the most effective strategies for diagnosing and treating CN PJI.

## Data Availability

The raw data supporting the conclusions of this article will be made available by the authors, without undue reservation.
