# Supplementary material for: Comprehensive analysis of culture-negative periprosthetic joint infection with metagenomic next-generation sequencing
Source: Front Cell Infect Microbiol. 2025 May 9;15:1564488. doi: 10.3389/fcimb.2025.1564488 (PMC12098451; doi:10.3389/fcimb.2025.1564488)
Supplement: Supplementary file 1 [file Table1.docx]

Supplementary table 1. Overview of the organisms identified in both groups.

| **Organism** | **CP PJI, n (%)** | **CN PJI, n (%)** |
| --- | --- | --- |
| **Common pathogens** | | |
| *Staphylococcus aureus* | 42 (19.4) | 8 (11.6) |
| *MSSA* | 28 (12.9) | 5 (7.2) |
| *MRSA* | 14 (6.5) | 3 (4.3) |
| *Staphylococcus epidermidis* | 44 (20.3) | 9 (13.0) |
| *Staphylococcus capitis* | 7 (3.2) | 2 (2.9) |
| *Streptococcus* | 15 (6.9) | 3 (4.3) |
| *Pseudomonas aeruginosa* | 7 (3.2) | 2 (2.9) |
| *Escherichia coli* | 8 (3.7) | 0 (0) |
| *Staphylococcus haemolyticus* | 8 (3.7) | 2 (2.9) |
| *Enterococcus faecalis* | 4 (1.8) | 1 (1.4) |
| *Staphylococcus hominis* | 2 (0.9) | 0 (0) |
| *Enterobacter cloacae* | 3 (1.4) | 0 (0) |
| *Klebsiella pneumoniae* | 9 (4.1) | 4 (5.8) |
| *Salmonella enterica* | 2 (0.9) | 2 (2.9) |
| *Staphylococcus pasteuri* | 1 (0.5) | 0 (0) |
| *Staphylococcus warneri* | 2 (0.9) | 0 (0) |
| *Staphylococcus lugdunensis* | 2 (0.9) | 0 (0) |
| *Acinetobacter nosocomialis* | 1 (0.5) | 1 (1.4) |
| *Propionibacterium acnes* | 1 (0.5) | 1 (1.4) |
| *Bacteroides fragilis* | 1 (0.5) | 0 (0) |
| **Rare pathogens** | | |
| *Mycoplasma* | 0 (0) | 5 (7.2) |
| *Candida albicans* | 4 (1.8) | 2 (2.9) |
| *Finegoldia magna* | 3 (1.4) | 2 (2.9) |
| *Candida parapsilosis* | 2 (0.9) | 3 (4.3) |
| *Candida tropical* | 3 (1.4) | 2 (2.9) |
| *Nontuberculous mycobacteria* | 0 (0) | 3 (4.3) |
| *Parvimonas micra* | 1 (0.5) | 2 (2.9) |
| *Enterococcus gallinarum* | 1 (0.5) | 0 (0) |
| *Elizabethkingia meningoseptica.* | 1 (0.5) | 0 (0) |
| *Stenotrophomonas maltophilia* | 0 (0) | 2 (2.9) |
| *Pseudomonas monteilii* | 0 (0) | 2 (2.9) |
| *Sphingomonas paucimobilis* | 0 (0) | 1 (1.4) |
| *Kocuria palustris* | 0 (0) | 1 (1.4) |
| *Coxiella burnetii* | 1 (0.5) | 1 (1.4) |

CP, culture positive; CN, culture negative; MRSA, methicillin-resistant S. aureus; MSSA, methicillin-susceptible S. aureus.
